# Network pharmacology combined with molecular docking and experimental validation to reveal the pharmacological mechanism of naringin against renal fibrosis

**DOI:** 10.1515/med-2023-0736

**Published:** 2023-06-08

**Authors:** Yanan Zhong, Xiang Li, Juan Xie, Yiyuan Zhang, Hailun Li, Donghui Zheng

**Affiliations:** Department of Nephrology, The Affiliated Huai’an Hospital of Xuzhou Medical University and Huai’an Second People’s Hospital, Huai’an, China

**Keywords:** naringin, renal fibrosis, network pharmacology, signaling pathway

## Abstract

To explore the pharmacological mechanism of naringin (NRG) in renal fibrosis (RF) based on network pharmacology combined with molecular docking and experimental validation. We used databases to screen for the targets of NRG and RF. The “drug-disease network” was established using Cytoscape. Gene Ontology (GO) and Kyoto Encyclopedia of Genes and Genomes (KEGG) analyses of targets were performed using Metascape, and molecular docking was performed using Schrödinger. We established an RF model in both mice and cells to validate the results of network pharmacology. After screening the database, we identified 222 common targets of NRG and RF and established a target network. Molecular docking showed that the target AKT had a good interaction with NRG. We found that the phosphatidylinositol 3-kinase (PI3K)/AKT signaling pathway was enriched by multiple targets and served as a target for experimental validation through GO and KEGG. The results revealed that NRG ameliorated renal dysfunction, reduced the release of inflammatory cytokines, decreased the expression of α-SMA, collagen I, and Fn, and recovered the expression of E-cad by inhibiting the PI3K/AKT signaling pathway. Our study used pharmacological analysis to predict the targets and mechanisms of NRG against RF. Furthermore, experiments proved that NRG inhibited RF effectively by targeting the PI3K/AKT signaling pathway.

## 1 Introduction

Chronic kidney disease (CKD) has gradually become a global public health problem. As of 2017, there were 697.5 million cases of CKD worldwide, and the number of deaths due to CKD is expected to reach 4 million by 2040 [1]. CKD leads to an increase in the global incidence rate and mortality, which seriously affects human health. Renal fibrosis (RF) is a common pathophysiological pathway for the progression of CKD to end-stage renal disease caused by various causes [2]. The main pathological features of RF are the continuous proliferation of renal interstitial fibroblasts, abnormal and excessive deposition of extracellular matrix (EMC), and excessive production of collagen I (COLI) and collagen III. This leads to the gradual destruction of renal tubular and interstitial structures until the complete loss of renal function [3,4]. The most important cause of RF is epithelial–mesenchymal transformation (EMT), which is characterized by renal tubular epithelial cells losing their epithelial phenotype, acquiring an immature mesenchymal phenotype, and activating myofibroblast (MFB) cells [5]. The skeletal proteins of MFB mainly include smooth muscle actin (α-SMA) and vimentin, among which MFB with positive expression of α-SMA is the main ECM synthesis cell that promotes the deposition of EMC and progressive renal interstitial fibrosis [6]. RF has become a global public health problem that seriously endangers human health [7]. Although it can be relieved by dialysis or kidney transplantation, the effect is limited. Most patients have poor prognoses and heavy economic burdens, making the treatment of RF a global challenge [8]. Exploring safe and efficient treatments for RF is crucial in the medical field. Compared to the high side effects of chemical drugs, natural plant compounds may become a new treatment for RF [9].

Naringin (NRG) is a dihydroflavonoid extracted from natural plants, which mainly exists in the immature dried exocarp of pomelo and citrus. Its chemical formula is C_27_H_32_O_14_, with a molecular weight of 580.53, and it is a light yellow or white-like powder with a bitter taste [10,11,12,13]. Pharmacological studies have shown that NRG has many biological activities, including antioxidant, anticancer, anti-inflammatory, antibacterial, and antifibrotic effects [14,15,16]. According to reports, NRG can be used in cardiovascular, metabolic, nervous, and respiratory diseases and plays a protective role in various pathological processes, such as liver fibrosis and pulmonary fibrosis [17,18]. In addition, NRG can effectively alleviate renal injury in kidney diseases caused by various factors. However, there are few studies on the role of NRG in RF. Therefore, it was imperative to determine whether NRG can alleviate RF and explore its underlying mechanism.

**Figure 1:**
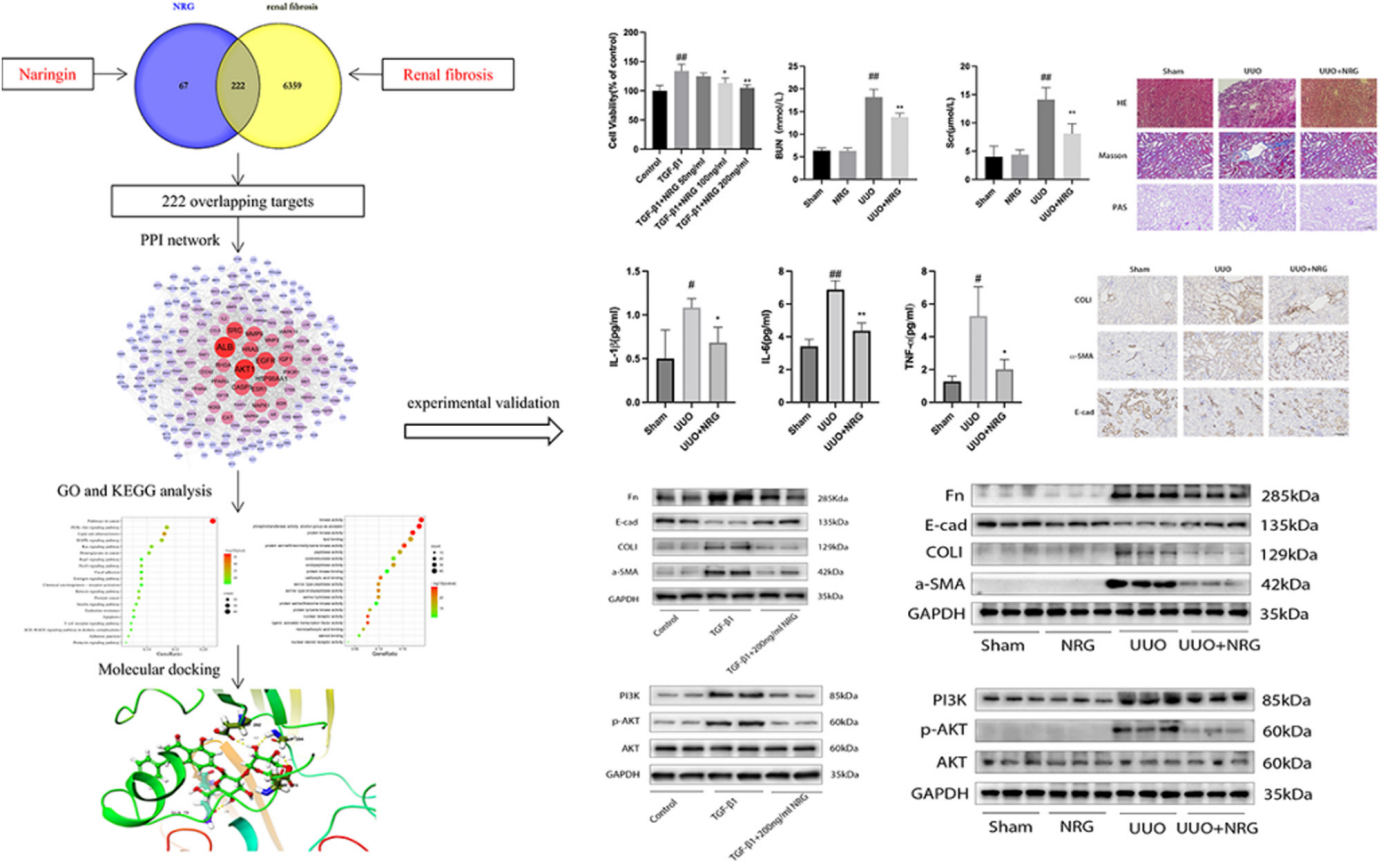
Flowchart for illustrating the mechanism of NRG against RF.

As a new field, network pharmacology is an interdisciplinary subject that combines systems biology and pharmacology [19]. Network pharmacology reflects the complex relationship between drugs and disease targets, revolutionizing the definition, diagnosis, treatment, and ideal cure of diseases [20]. Network pharmacology emphasizes the multi-channel regulation of signal pathways, improves the therapeutic effect of drugs, and reduces toxicity and side effects, thus improving the treatment success rate and saving the cost of drug research and development [21]. Molecular docking is a method based on a silicon-based structure widely used in drug discovery to identify new compounds with therapeutic significance and predict ligand–target interactions at the molecular level [22]. Our study used network pharmacology methods combined with molecular docking and experimental validation to explore the main targets and molecular biological mechanisms of NRG in the treatment of RF to provide a scientific theoretical basis for follow-up clinical application of NRG. A flowchart of the study is shown in Figure 1.

## 2 Materials and methods

### 2.1 Network pharmacology

#### 2.1.1 Screening the targets of NRG and RF

Type “Naringin” into the PubChem database to search and then click on the Structure column to download the 3D chemical structure of NRG. (https://pubchem.ncbi.nlm.nih.gov/compound/442428) [23]. Based on the 3D structure of NRG, the potential targets of NRG are screened by the PharmMapper database (http://www.lilab-ecust.cn/pharmmapper/) and the targets were converted into official names through the UniProt database (https://www.uniprot.org/) [24,25,26]. Screen potential targets of RF in GeneCards database through the keyword “RF” (https://www.genecards.org/).

#### 2.1.2 Construction of drug–target disease network

The common targets of NRG and RF were determined using a Venn diagram (https://bioinfogp.cnb.csic.es/tools/venny/index.html) [27], which was imported into Cytoscape to construct a drug–target disease network.

#### 2.1.3 Construction of PPI Network

The common targets of NRG and RF were input into STRING (https://string-db.org/) to obtain protein interaction information, which was imported into Cytoscape to construct a protein–protein interaction (PPI) network. Then, we performed a network analysis of the PPI network and screened the top 10 targets according to the degree centrality (DC) values.

#### 2.1.4 Functional enrichment analysis

Metascape (https://metascape.org/) [28] was used to perform Gene Ontology (GO) and KEGG functional enrichment analyses of the core targets. The GO and KEGG pathway analyses were screened for *P* < 0.05, and the top 20 GO and KEGG analyses were mapped as bubble plots. The top 20 results of KEGG were imported into Cytoscape to obtain the network of the “target - KEGG enrichment pathway.”

### 2.2 Molecular docking

We used NRG as a ligand, selected some key targets as receptors, and docked them using Schrödinger software. First, the 3D structure of NRG was obtained from PubChem, and the ligand structure was optimized using the LigPrep module. The protein sequence of the key target receptor protein was determined using the PDB database (https://www.rcsb.org), and the receptor protein was optimized using the Protein Preparation Wizard module. Finally, molecular docking was carried out using the Ligand Docking module, and the results were visualized using PyMOL software [29].

### 2.3 Main reagents and kits

NRG was purchased from Sigma-Aldrich (CAS No. 71162; purity ≥95%). Dulbecco’s modified eagle medium and 0.25% Trypsin-EDTA were purchased from Gibco. Penicillin–streptomycin was purchased from BasalMedia. Fetal bovine serum was purchased from EXcell Bio. Dimethyl sulfoxide (DMSO) was purchased from Sevier Bio. Cell Counting Kit-8 (CCK-8) was purchased from Yeasen Biotechnology (Shanghai, China). TGF-β1 was purchased from GenScript Inc. Radioimmunoprecipitation assay (RIPA) buffer, phenylmethylsulfonyl fluoride, 50× cocktail protease inhibitor, phosphorylated protease inhibitor, and BCA protein quantitative detection kit were purchased from Servicebio. α-SMA, E-cad, phosphatidylinositol 3-kinase (PI3K), AKT, and p-Akt were purchased from Cell Signaling Technology. COLI was purchased from Affinity Biosciences. Fn was purchased from Abcam. Horseradish peroxidase goat anti-rabbit IgG (H + L) was purchased from ABclonal. The PAGE Gel Fast Preparation Kit was purchased from Epizyme. GAPDH, a Marker (10–180 kDa), and Super ECL Plus were purchased from PROTEINBIO.

### 2.4 Experimental validation

#### 2.4.1 *In vitro* study

Mouse renal tubular epithelial (mRTE) cells were seeded in 96-well plates at a density of 5 × 10^3^ cells/well and incubated with different concentrations of NRG for 48 h. CCK-8 was used to calculate cell viability. Cell viability = (absorbance of treatment group − absorbance of blank)/(absorbance of control group − absorbance of blank) × 100%. mRTE cells were seeded into 96-well plates at 1×10^4^ cells/well density. When the cell density reached 40–50%, 10 ng/mL TGF- β1 was added to the control group, and 10 ng/mL TGF-β1 and different concentrations of NRG were added to the treatment group for 48 h. The cell survival rate was also calculated using CCK-8, and the cells were collected for subsequent WB experiments.

#### 2.4.2 *In vivo* study

##### 2.4.2.1 Animal experiments

Male C57BL/6J mice (8 weeks old, weighing 20–22 g) were purchased from Jiangsu Jicui Yaokang Biotechnology Co., Ltd. and housed in a standard laboratory animal facility with a 12 h light/dark cycle, 20°C, 55% relative humidity, and free access to food and water. Unilateral ureteral obstruction (UUO) animal models have been widely used to simulate pathological changes in RF. Within 7 days of modeling, the kidneys of UUO mice showed apparent changes in hemodynamics and metabolism [30]. After anesthesia, a longitudinal incision was made on the left back to expose the left kidney and ureter, and the left ureter was dissociated. After ligation with 5-0 silk thread, the back was sutured layer by layer. Mice were randomly divided into four groups: sham (*n* = 5), NRG (*n* = 5), UUO (*n* = 5), and UUO + NRG (*n* = 5). In the sham and NRG groups, the left ureter was dissociated without ligation. Mice in the NRG and UUO + NRG groups were intraperitoneally injected with NRG 100 mg/kg on the second day after modeling. The sham and UUO groups were administered the same volume of 0.1% DMSO for 7 days. Animals were sacrificed 7 days later to collect renal tissue and blood samples for subsequent experiments.

##### 2.4.2.2 Renal function and inflammatory factors

The levels of blood urea nitrogen (BUN) and serum creatinine (Scr) were measured using commercial kits according to the manufacturer’s instructions (Jiancheng Bioengineering Institute, Nanjing, China). The concentrations of inflammatory factors (TNF-α, IL-1β, and IL-6) were measured using an enzyme-linked immunosorbent assay kit according to the manufacturer’s instructions (Elabscience, Wuhan, China).

##### 2.4.2.3 Histological and immunohistochemical staining

Kidneys were fixed with 4% paraformaldehyde, embedded in paraffin, and sliced into 5-µm-thick sections. Hematoxylin and eosin (HE), Masson, and PAS staining kits (Sevier, Wuhan, China) were used for histological staining. For immunohistochemical staining, each sample was incubated with primary antibodies COLI (1:100), E-Cad (1:400), and α-SMA (1:500) at 4°C overnight. The renal tubular injury scale (0, no injury; 1, mild injury; 2, moderate injury; 3, severe injury) was used as the semi-quantitative score. Image-Pro Plus software (version 6.0) was used to calculate the percentage of positive areas in each experimental group for statistical analysis (percentage positive area = positive area/tissue area × 100%).

##### 2.4.2.4 Western blot analysis

Total protein was extracted from the RIPA lysate and quantified using the bicinchoninic acid method. The proteins were separated by sodium dodecyl sulfate-polyacrylamide gel electrophoresis and transferred to an nitrocellulose filter membrane. Membranes were incubated with primary antibodies against COLI (1:1,000), Fn (1:1,000), E-cad (1:1,000), α-SMA (1:1,000), PI3K (1:1,000), AKT (1:1,000), p-AKT (1:1,000), and GAPDH (1:1,000) at 4°C overnight. The results were visualized using Super ECL Plus and developed using a gel imaging system (Azure Biosystems).


**Institutional review board statement:** The studies involving animals were reviewed and approved by the Ethics Committees of The Affiliated Huai’an Hospital of Xuzhou Medical University and Huai’an Second People’s Hospital.

## 3 Results

### 3.1 Network pharmacology

#### 3.1.1 The common targets of NRG and RF

**Figure 2:**
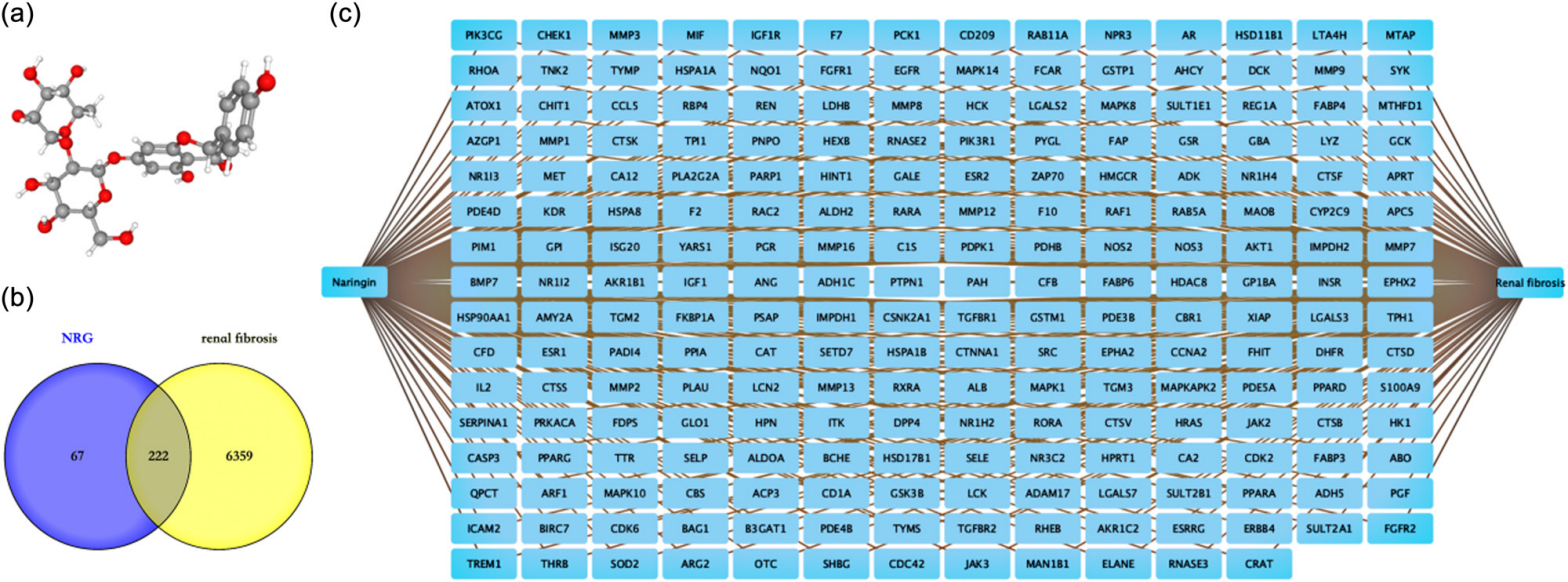
The common targets of NRG and RF. (a) NRG chemical structure. (b) Venn diagram of NRG and RF common targets. (c) NRG–target RF network.

Based on the three-dimensional chemical structure of NRG (Figure 2a), we used public databases to predict the potential targets and RF target genes, including 289 NRG and 6,581 RF. We identified 222 common targets that cross-targeted NRG and RF (Figure 2b) and constructed a drug–target disease network (Figure 2c).

#### 3.1.2 PPI network and core targets

**Figure 3:**
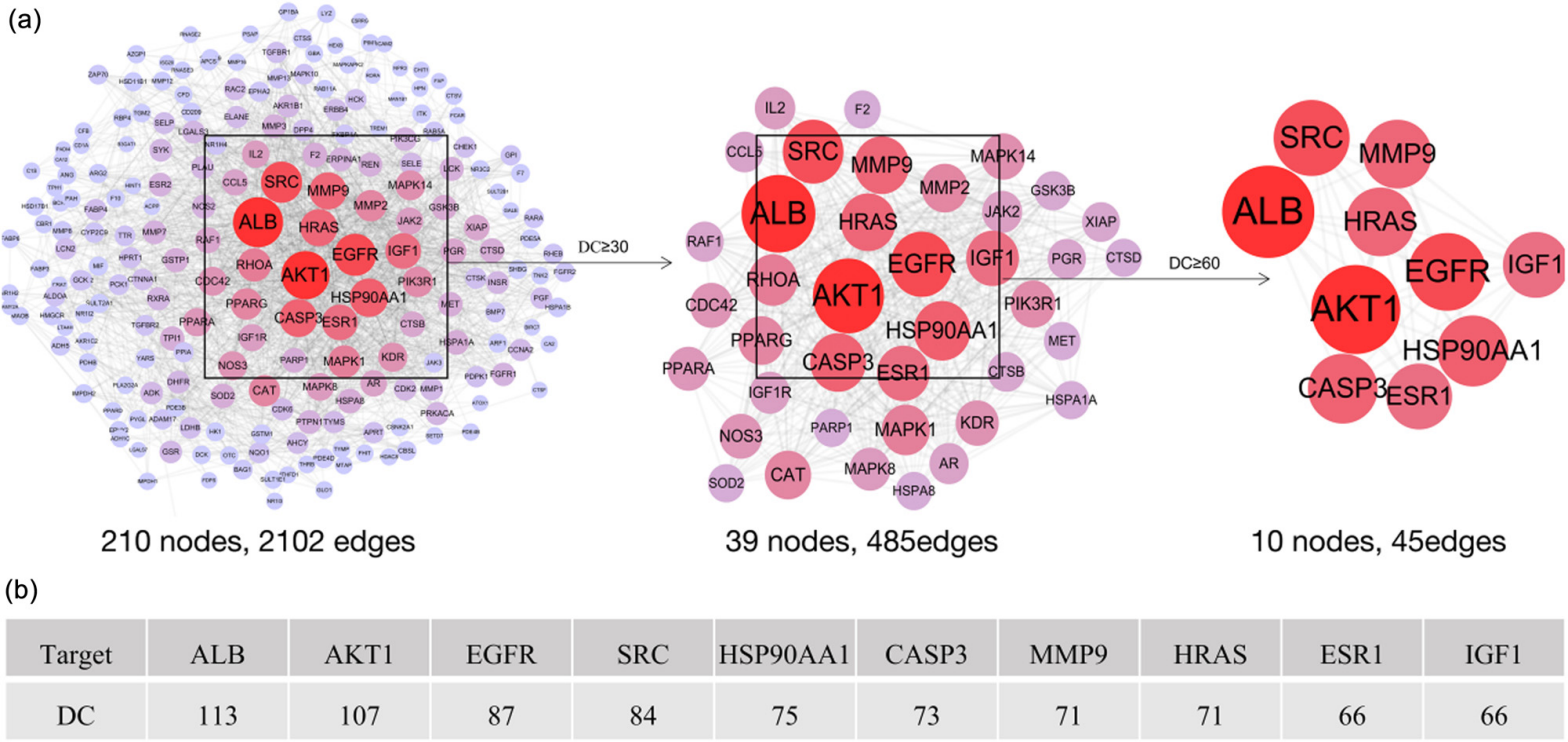
PPI network and core targets. (a) PPI network. (b) Top 10 core targets.

The common targets of NRG and RF were inputted into STRING, and a PPI network with 210 nodes and 2,102 edges was constructed using Cytoscape (Figure 3a). The size of the nodes in the PPI network was adjusted according to their degree. The color gradient was adjusted by betweenness centrality. Nodes that are larger and redder indicate higher correlations with other proteins in the network, indicating greater importance. Then, we performed a network analysis of the PPI network and screened the top 10 targets according to the DC values (Figure 3b).

#### 3.1.3 GO and KEGG enrichment analysis

**Figure 4:**
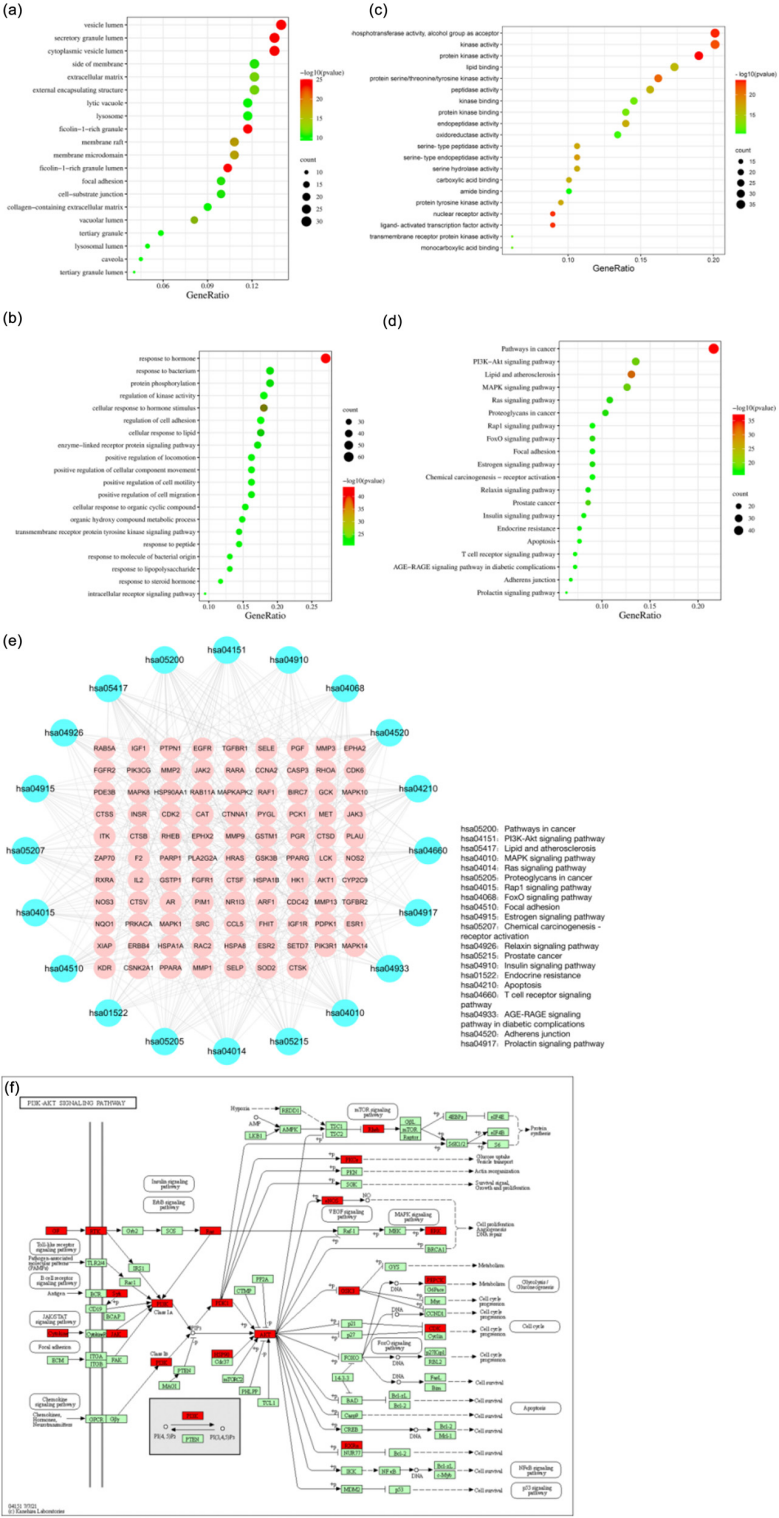
GO and KEGG enrichment analysis. (a) The top 20 entries of BP. (b) The top 20 entries of CC. (c) The top 20 entries of MF. (d) The top 20 entries of KEGG. (e) NRG against RF targets–KEGG enrichment pathway. (f) PI3K/AKT signaling pathway.

GO and KEGG functional enrichment analyses were carried out to investigate the biological processes or potential signaling pathways regulated by NRG. GO enrichment analysis showed that 1,607 biological progress (BP), 84 cellular components (CC), and 190 molecular functions (MF) were obtained using Metascape; the top 20 entries of BP, CC, and MF are shown in Figure 4a–c. BP analysis showed that the common targets were mainly focused on the response to hormones, regulation of kinase activity, and protein phosphorylation. CC analysis was mainly focused on the vesicle lumen, secretory granule lumen, cytoplasmic vesicle lumen, etc. In addition, MF was primarily enriched in kinase activity, phosphotransferase activity, and protein kinase activity. Metascape was also used for KEGG analysis, and 200 results were obtained. The 20 most significant KEGG results related to NRG are shown in Figure 4d. The top 20 KEGG results of KEGG into Cytoscape to obtain the network of “target–KEGG enrichment pathway” (Figure 4e). In addition, there were 43 signaling pathways related to our profession, of which the PI3K/AKT signaling pathway ranked first, and its predictive targets are shown in detail in Figure 4f.

### 3.2 Molecular docking

**Figure 5:**
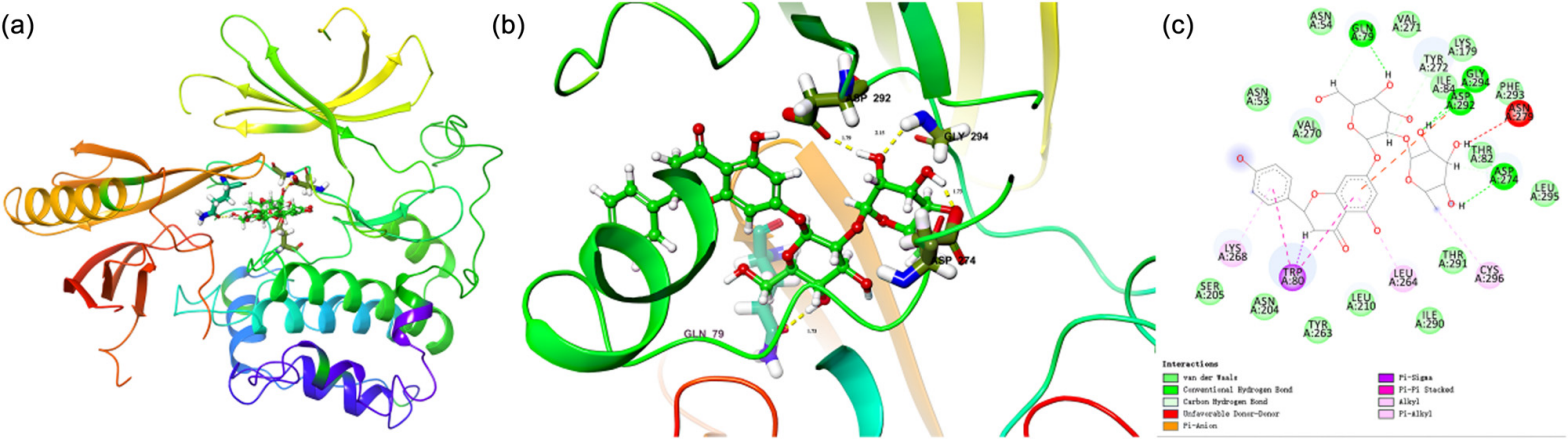
Molecular docking. (a) Overall 3D map. (b) Active pocket diagram. (c) 2D action diagram.

According to the screened core targets and the results of KEGG analysis, NRG most likely affected RF by regulating the PI3K/AKT signaling pathway. Therefore, we used NRG as the ligand and AKT1 (PDB database number: 7NH5) as the receptor to carry out molecular docking using Schrödinger and visualization using PyMOL, as shown in Figure 5. It is generally believed that the lower the binding energy, the higher the affinity between the receptor and ligand, and the greater the possibility of action. Our molecular docking results showed that the binding energies of NRG and AKT1 were −9.964 kcal/mol, which indicated that NRG and AKT1 had high docking activity and affinity.

### 3.3 Experimental validation

#### 3.3.1 *In vitro* study

**Figure 6:**
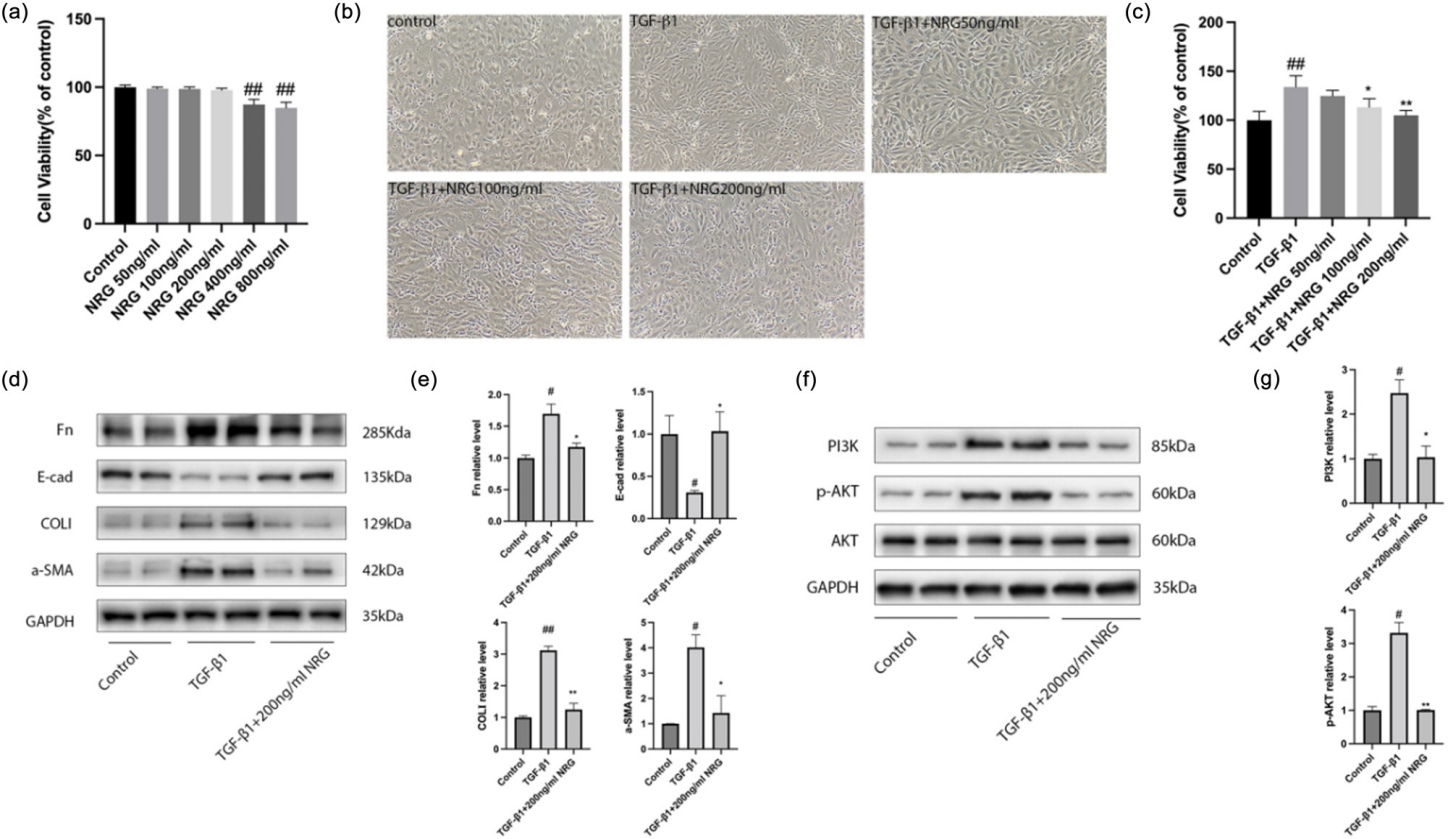
*In vitro* study. (a) Cell viability of each group mRTE cells. (b) Morphological changes in mRTE (×100). (c) Cell viability of different group cells. (d) Representative Western blot for Fn, COLI, a-SMA, and E-cad. (e) The relative protein levels of Fn, COLI, a-SMA, and E-cad. (f) Representative Western blot for PI3K and p-AKT. (g) The relative protein levels of PI3K and p-AKT. Data are presented as mean ± SD, ^#^ *P* < 0.05 vs control, ^##^ *P* < 0.01 vs control, ^*^ *P* < 0.05 vs TGF-β1, ^**^ *P* < 0.01 vs TGF-β1.

First, at diluted concentrations 50, 100, and 200 ng/mL, NRG showed no obvious cytotoxicity to mRTE cells, while the concentration reached 400 ng/mL, and the cell viability decreased significantly (Figure 6a). It can be seen that the maximum safe concentration of NRG to mRTE cells is 200 ng/mL. We found that the morphology of cells stimulated with 10 ng/mL TGF-β1 for 48 h changed, showing an elongated spindle shape (Figure 6b), and the number of cells increased significantly. However, NRG at concentrations 100 and 200 ng/mL inhibited the effects of TGF-β1 on cells (Figure 6c). We also found that NRG at a diluted concentration of 200 ng/mL inhibited the expression of α-SMA, COLI, and Fn and promoted the expression of E-cad (Figure 6d and e). Furthermore, the expression of PI3K and p-AKT in the TGF-β1 group was elevated, whereas the levels of PI3K and p-AKT in cells incubated with NRG were dramatically reduced (Figure 6f and g). These results indicate that NRG can effectively suppress RF *in vitro* by inhibiting the PI3K/AKT signaling pathway.

#### 3.3.2 *In vivo* study

##### 3.3.2.1 Renal function and inflammatory factors

**Figure 7:**
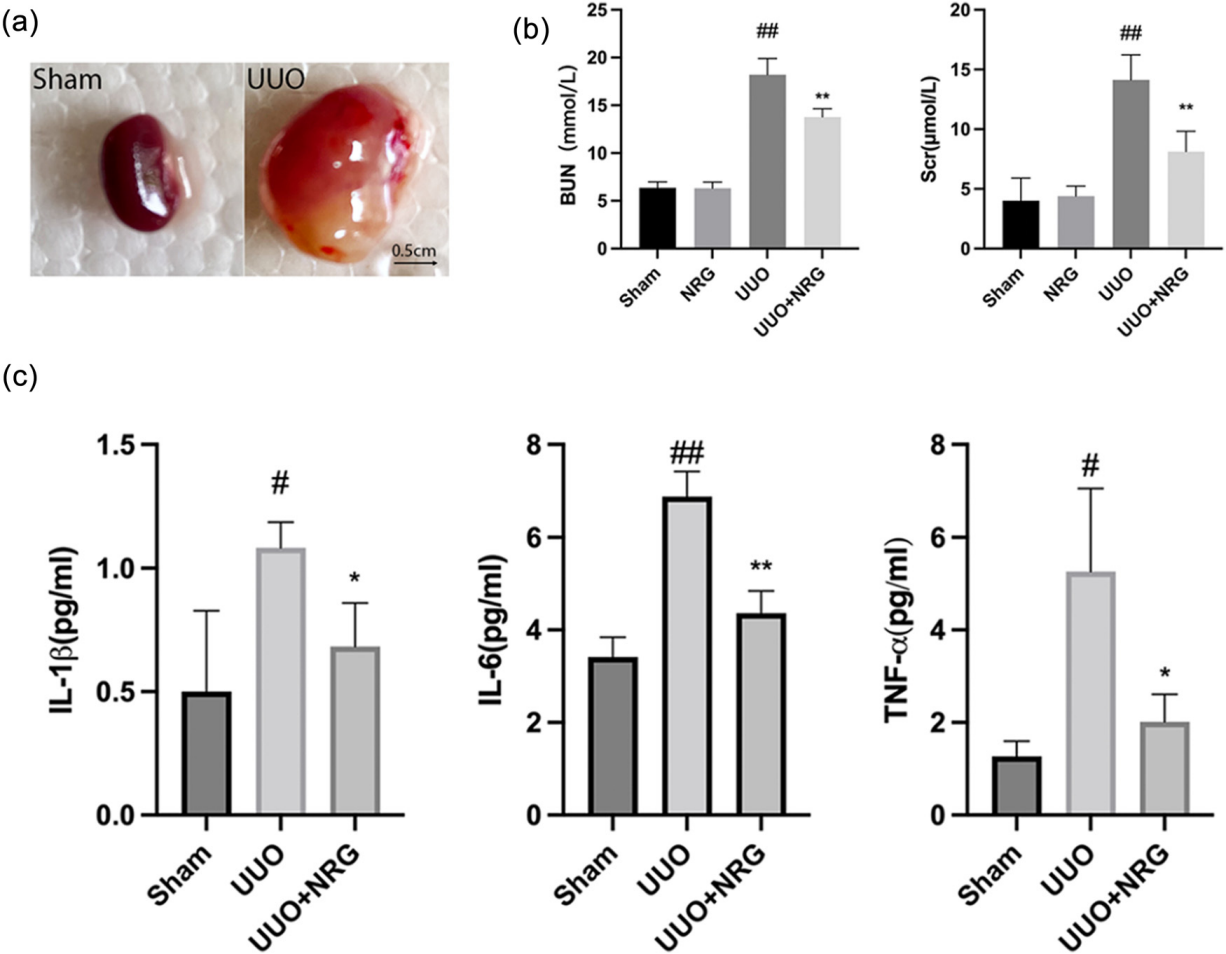
Renal function and inflammatory factors. (a) The naked eye volume of the mouse kidney. (b) BUN and Scr level. (c) The levels of IL-1β, IL-6, and TNF-α. Data are presented as mean ± SD, ^#^ *P* < 0.05 vs sham, ^##^ *P* < 0.01 vs sham, *P < 0.05 vs UUO, **P < 0.01 vs UUO.

In the UUO group, the naked eye volume of the mouse kidney was significantly increased, and there was a urinary vesicle containing yellow turbid urine, indicating that the model was successful (Figure 7a). Renal damage in UUO mice led to a sharp increase in BUN and Scr (Figure 7b). After NRG treatment, the levels of IL-1 β, IL-6, and TNF-α decreased significantly (Figure 7c), indicating that NRG reduced renal damage and inflammatory response to RF.

##### 3.3.2.2 Histological and immunohistochemical staining

**Figure 8:**
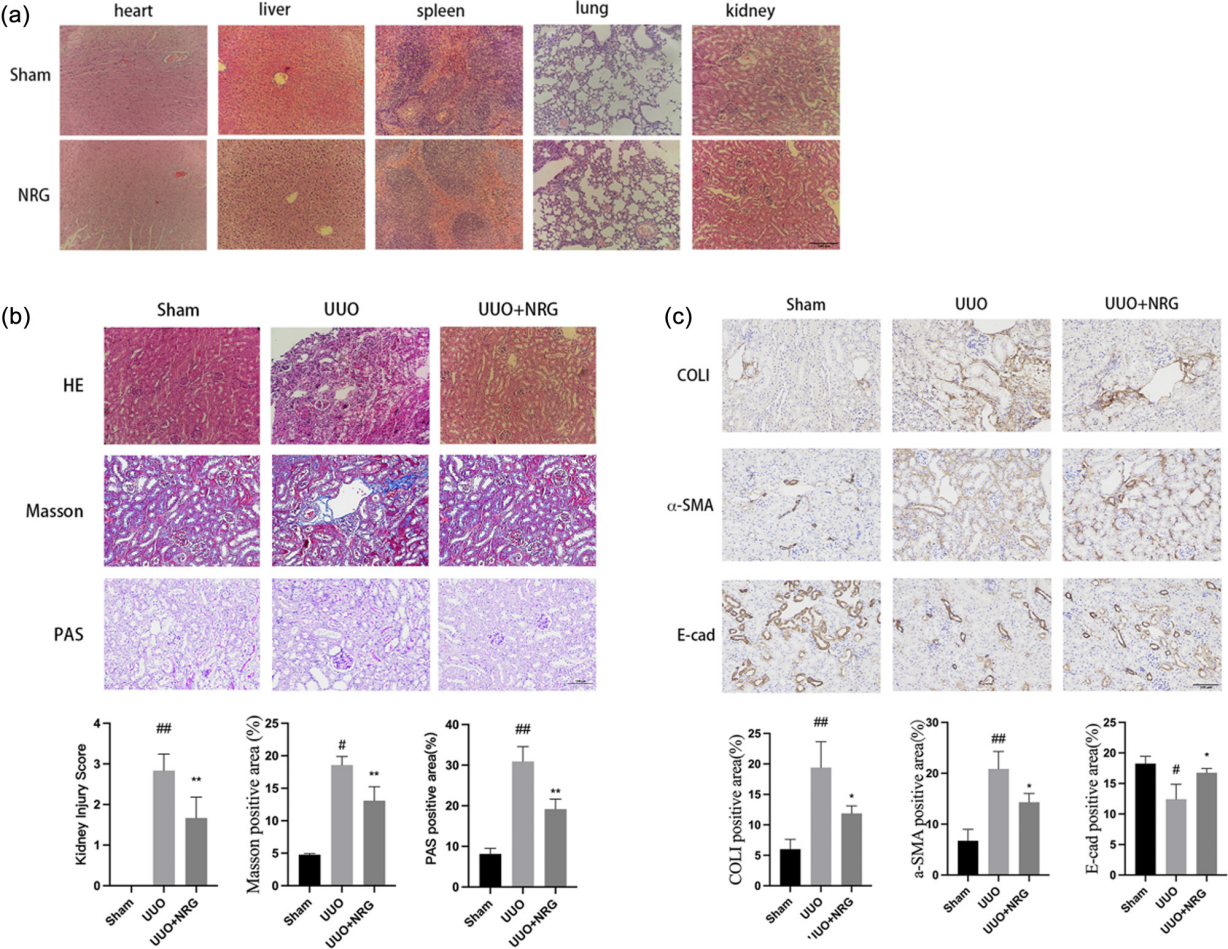
Histological and immunohistochemical staining. (a) HE of organs in sham and NRG. (b) HE, Masson, and PAS map and semi-quantitative score/positive area (%) of each group. (c) Immunohistochemical map and positive area (%) of each group. Magnification: ×200. Scale bar: 100 μm. Data are presented as mean ± SD, ^#^ *P* < 0.05 vs sham, ^##^ *P* < 0.01 vs sham, *P < 0.05 vs UUO, **P < 0.01 vs UUO.

HE results showed that the structures of the heart, liver, spleen, lung, and kidney of the mice in the NRG group were normal (Figure 8a), which was not different from that of the sham group, indicating that NRG had no toxicity or damage to various organs of mice. The renal tubular structure in the sham group appeared normal, while the UUO group showed highly edematous renal tubular epithelial cells and diffuse infiltration of inflammatory cells (Figure 8b). PAS showed that the glomerular basement membrane and interstitium of the mice in the UUO group were significantly proliferated, the lumen of the renal tubules was dilated, and the brush border disappeared. After NRG treatment, the renal injury was significantly reduced compared with UUO (Figure 8b). The kidneys were stained with Masson’s trichrome to capture collagen accumulation. UUO showed obvious collagen fiber formation, while the collagen was significantly reduced after NRG treatment, and the positive area was smaller than that of UUO (Figure 8b). In addition, after NRG treatment, immunohistochemical results showed that COLI and α-SMA expression decreased and E-cad expression returned to normal compared with UUO (Figure 8c), which further confirmed that NRG significantly inhibited RF.

### 3.4 Western blot analysis

**Figure 9:**
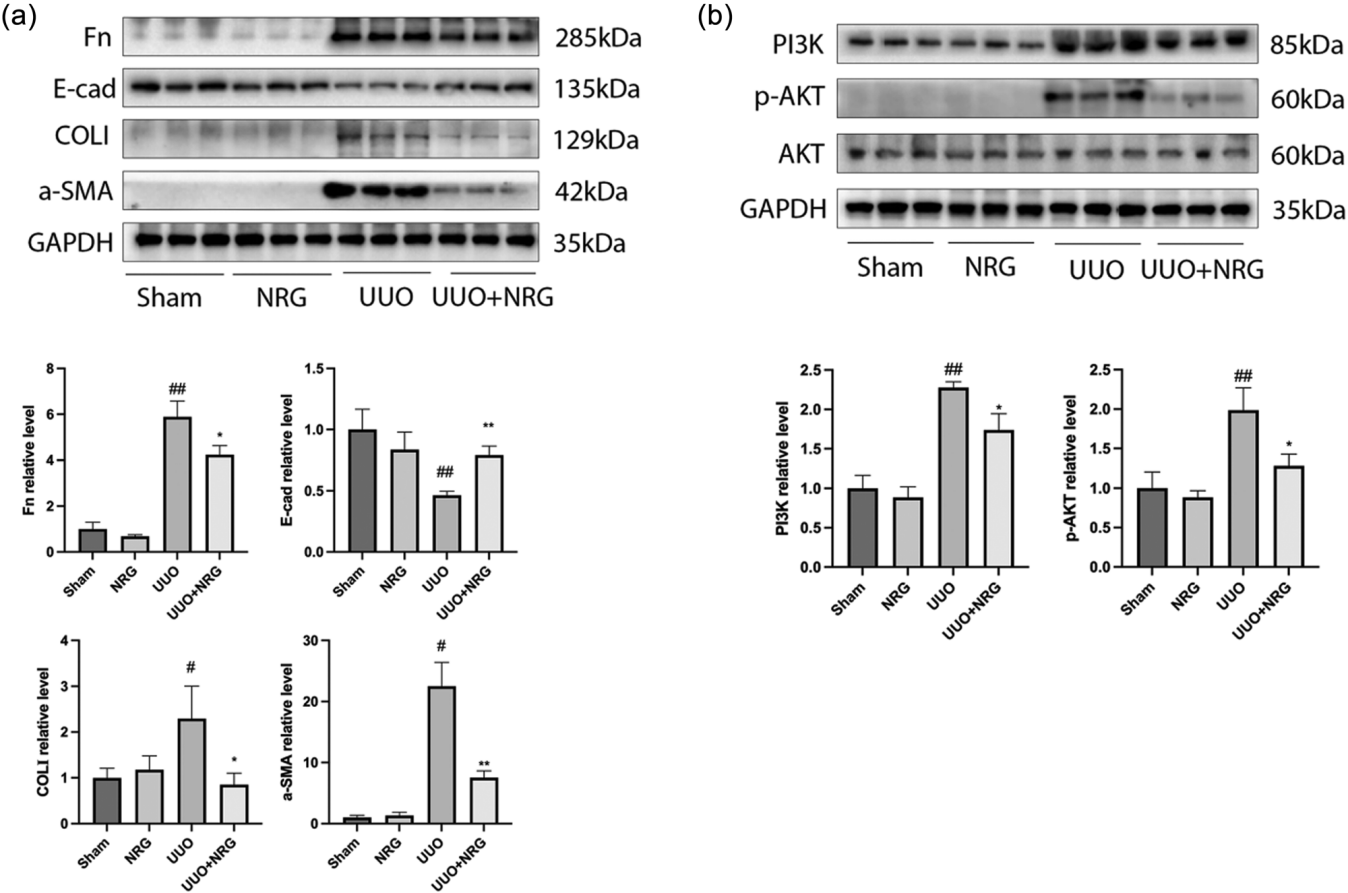
(a) Western blot and relative protein levels of Fn, COLI, a-SMA, and E-cad. (b) Western blot and relative protein levels of PI3K and p-AKT. Data are presented as mean ± SD, ^#^ *P* < 0.05 vs sham, ^##^ *P* < 0.01 vs sham, *P < 0.05 vs UUO, **P < 0.01 vs UUO.

The expression of α-SMA, COLI, and Fn in UUO was significantly higher than that in the sham group, while the expression of E-cad was significantly lower than that in the sham group. After NRG treatment, the upregulation of α-SMA, COLI, and Fn was inhibited, and E-cad expression was promoted (Figure 9a). The expression of PI3K and p-AKT in UUO was elevated (Figure 9b), whereas the levels of PI3K and p-AKT in UUO + NRG were dramatically reduced. These results indicate that NRG suppressed RF *in vivo* by inhibiting the PI3K/AKT signaling pathway.

## 4 Discussion

RF is an irreversible pathological change in the progression of CKD to ERSD. The pathogenesis and development of RF involve many mechanisms, including a variety of factors related to the activation of various cells, cytokines, and vasoactive substances, which have not been fully elucidated [31,32]. As a flavonoid compound extracted from plants, NRG has a variety of biological activities. Many studies have shown that flavonoids can treat kidney diseases by inhibiting inflammatory reactions, apoptosis, and regulating signaling pathways [33]. Network pharmacology is a novel method for systematically detecting the mechanisms of action of Chinese herbal medicine [34]. Our study investigated the effect of NRG on RF and its possible mechanism based on network pharmacology combined with experimental validation.

We first described 222 potential targets of NRG in the treatment of RF through network pharmacology, and the top 10 key targets selected according to their degree included ALB, AKT1, EGFR, SRC, and HSP90AA1. AKT1 is one of the three mammalian subtypes of AKT kinase, a serine/threonine kinase activated by PI3K [35]. Studies have shown that AKT1 is involved in the proliferation and activation of interstitial fibroblasts, mesangial cells, and tubular epithelial cells during RF development [36]. Inhibiting the activation of AKT1 can inhibit the inflammatory response, fight renal injury, and slow the progression of CKD [37]. EGFR is a family of epidermal growth factor receptors expressed in large amounts along the nephron in the kidney [38]. When EGFR ligand is added to renal tubular cells *in vitro*, it can promote cell proliferation, mesenchymal–epithelial cell transdifferentiation, and collagen production and plays a major role in developing renal diseases [39]. It can be seen that NRG suppression of RF is achieved through multiple targets.

The mechanism of action of NRG against RF remains unknown. Therefore, we used network pharmacology to explore the possible pharmacological mechanism. The results of the KEGG enrichment analysis showed that the related targets were mainly enriched in PI3K–AKT, MAPK, RAS, and other signaling pathways, which may be the key signaling pathways of NRG against RF. PI3K is a group of plasma membrane-related lipoproteins that catalyzes the production of phosphatidylinositol triphosphate, resulting in AKT binding to the cell membrane [40]. PI3K inhibitor and PI3K knockout short hairpin RNA can block AKT phosphorylation and reduce oxidative stress and inflammation [41]. The MAPK cascade is a key signaling pathway for regulating various cellular processes. Zhou et al. [42] showed that inhibition of the MAPK pathway could reduce the expression of pro-inflammatory factors, inhibit renal cell apoptosis, and down-regulate TGF-β1 expression to alleviate RF. Therefore, we suspect that NRG can alleviate RF through multiple signaling pathways.

The classic pro-inflammatory cytokines IL-6, IL-1β, and TNF-α have the effect of promoting fibrosis, which has been observed in fibrosis models of lung, heart, kidney. and other organs [43]. In this study, the levels of IL-6, IL-1β, and TNF-α in the UUO group were significantly higher than those in the sham group, but the levels of inflammatory cytokines in the NRG group were significantly lower than those in the UUO group, which indicated that NRG could relieve fibrosis by reducing the inflammatory response. Fn and COLI are the main components of ECM. Excessive accumulation of ECM will promote renal parenchyma sclerosis and fibrous scar formation, thus destroying normal renal tissue [44]. The results showed that the expression of Fn and COLI in the UUO group was higher than that in the sham group, but the expression of Fn and COLI in the UUO + NRG group was lower than that in the UUO group, which indicated that NRG could reduce the expression of Fn and COLI and prevent the excessive accumulation of ECM.

In the process of EMT, epithelial cells reduce adhesion markers such as E-cad and obtain interstitial markers such as α-SMA, thus losing the characteristics of cell adhesion [45]. The results showed that the expression of α-SMA in the UUO group was higher than that in the sham group, while the expression of E-cad was lower than that in the sham group. However, after treatment with NRG, the expression of α-SMA was significantly lower than that of the UUO group, and the expression of E-cad was higher than that of the UUO group, which indicated that NRG could reduce the expression of α-SMA, restore E-cad expression, and inhibit the process of EMT. In addition, EMT is closely related to the activation of the PI3K/AKT signal pathway [46]. In this experiment, the expression of PI3K and p-AKT in the UUO group was higher than that in the sham group, but the expression of PI3K and p-AKT in the UUO + NRG group was significantly lower than that in the UUO group. This confirms that NRG can inhibit EMT and slow down the progression of fibrosis by inhibiting the activation of the PI3K/AKT signal pathway.

In this study, the related targets and signal pathways of NRG in the treatment of RF were obtained for the first time through network pharmacological analysis. It was verified by experiments that NRG can promote the recovery of renal function, reduce renal tissue injury, reduce inflammatory reaction, and inhibit PI3K/AKT pathway to reduce fibrosis. Our research findings undoubtedly enrich the pharmacological mechanisms of RF, provide a new option for the treatment of fibrosis, and provide a theoretical basis for the use of natural drugs for disease treatment. However, there are also some shortcomings in our research: one is that the database used in network pharmacological analysis may be lack of completeness and accuracy and the other is that we only carry out experimental verification in cell and mouse models. the relevant data of clinical patients were not collected.

## 5 Conclusion

Our study predicted the targets and potential mechanism of NRG in treating RF through network pharmacology and preliminarily clarified that NRG may treat RF through multiple targets and pathways. In addition, we verified that NRG could alleviate RF by inhibiting the PI3K/AKT signaling pathway *in vitro* and *in vivo*, providing a scientific basis for further research and clinical application.
